# PCA3 and PCA3-Based Nomograms Improve Diagnostic Accuracy in Patients Undergoing First Prostate Biopsy

**DOI:** 10.3390/ijms140917767

**Published:** 2013-08-29

**Authors:** Alain Ruffion, Marian Devonec, Denis Champetier, Myriam Decaussin-Petrucci, Claire Rodriguez-Lafrasse, Philippe Paparel, Paul Perrin, Virginie Vlaeminck-Guillem

**Affiliations:** 1 Department of Urology, University Hospital of Lyon Sud, Hospices Civils of Lyon, Pierre Bénite 69495, France; E-Mails: alain.ruffion@chu-lyon.fr (A.R.); marian.devonec@chu-lyon.fr (M.D.); denis.champetier@chu-lyon.fr (D.C.); philippe.paparel@chu-lyon.fr (P.P.); paul.perrin@chu-lyon.fr (P.P.); 2 Medical Faculty of Lyon 1 University, Lyon 69000, France; E-Mails: myriam.decaussin-petrucci@chu-lyon.fr (M.D.-P.); claire.rodriguez-lafrasse@chu-lyon.fr (C.R.-L.); 3 Department of Pathology, University Hospital of Lyon Sud, Hospices Civils of Lyon, Pierre Bénite 69495, France; 4 Medical Unit of Molecular Oncology and Transfer, Department of Biochemistry and Molecular biology, University Hospital of Lyon Sud, Hospices Civils of Lyon, Pierre Bénite 69495, France

**Keywords:** urine biomarker, nomogram, initial prostate biopsy, prostate cancer, prostate cancer antigen 3

## Abstract

While now recognized as an aid to predict repeat prostate biopsy outcome, the urinary PCA3 (prostate cancer gene 3) test has also been recently advocated to predict initial biopsy results. The objective is to evaluate the performance of the PCA3 test in predicting results of initial prostate biopsies and to determine whether its incorporation into specific nomograms reinforces its diagnostic value. A prospective study included 601 consecutive patients addressed for initial prostate biopsy. The PCA3 test was performed before ≥12-core initial prostate biopsy, along with standard risk factor assessment. Diagnostic performance of the PCA3 test was evaluated. The three available nomograms (Hansen’s and Chun’s nomograms, as well as the updated Prostate Cancer Prevention Trial risk calculator; PCPT) were applied to the cohort, and their predictive accuracies were assessed in terms of biopsy outcome: the presence of any prostate cancer (PCa) and high-grade prostate cancer (HGPCa). The PCA3 score provided significant predictive accuracy. While the PCPT risk calculator appeared less accurate; both Chun’s and Hansen’s nomograms provided good calibration and high net benefit on decision curve analyses. When applying nomogram-derived PCa probability thresholds ≤30%, ≤6% of HGPCa would have been missed, while avoiding up to 48% of unnecessary biopsies. The urinary PCA3 test and PCA3-incorporating nomograms can be considered as reliable tools to aid in the initial biopsy decision.

## 1. Introduction

The widespread use of the prostate-specific antigen (PSA) test proved to improve early diagnosis of prostate cancer (PCa) [1]. The PSA test is nevertheless characterized by a poor specificity. Non-malignant prostate pathologies, such as prostatitis, or benign prostate hyperplasia can also induce increased PSA levels, resulting in a high proportion (up to 70%) of negative and eventual unnecessary prostate biopsies. The poor PSA specificity also led to the overdiagnosis and, potentially, the overtreatment of indolent PCas that do not evolve towards aggressive life-threatening cancers [1]. Many efforts are consequently made to develop new biomarkers that could complement PSA for early PCa diagnosis. Urinary detection of prostate cancer gene 3 (PCA3), developed for a decade [2], is a useful adjunct in predicting prostate biopsy outcome [3,4], particularly for patients with previous negative biopsies [5]. In fact, studies have often included patients scheduled for either repeat or initial biopsies [2,6–11]. Whether the PCA3 test could also be useful in guiding the initial biopsy decision [3] has been specifically addressed only recently, with convincing results [12–15].

Nomograms are widely used to help physicians in decision guiding and proved to be more accurate than the separate use of a marker. When reporting diagnostic performances of the urinary PCA3 test, some authors also compared the diagnostic accuracy of base nomograms and nomograms, including the PCA3 score. The addition of the PCA3 score always gave better accuracy, suggesting that the PCA3 score is a strong independent predictor of biopsy results and should, therefore, be included in nomograms [9,12,16–19]. To our knowledge, only four urinary PCA3-based nomograms have been previously published. Two are proposed to all patients, whatever the medical history of previous biopsies, and were externally validated: the updated version of the Prostate Cancer Prevention Trial (PCPT) risk calculator (online available) and the graphically available nomogram published by Chun *et al.* [20–22]. Another is specifically dedicated to patients scheduled for repeat biopsy [23], while the last one, very recently published by Hansen *et al.* [14], has been developed for guiding the initial biopsy decision. Both Hansen’s and Chun’s nomograms proved to provide significant clinical benefit without missing a too important proportion of high-grade prostate cancer (HGPCa) [14,21].

In this study, we therefore aimed to (1) evaluate the diagnostic performance of the urinary PCA3 test to predict the outcome of initial prostate biopsies; and (2) perform a head-to-head comparison of the three urinary PCA3-based nomograms currently available for initial or mixed biopsy patients.

## 2. Results and Discussion

### 2.1. Characteristics of Our Validation Cohort

Urine samples were obtained from 601 consecutive patients addressed for initial prostate biopsy and, 594 samples were informative for the PCA3 test (99%). Patients’ characteristics are summarized in Table 1. Positive biopsies were observed in 276 patients (46%), including 128 patients with HGPCa (Gleason score ≥ 7), *i.e.*, 46% of all prostate cancer (PCa) diagnosed. See Table S1 for additional pathological findings.

### 2.2. Diagnostic Performance of the Urinary PCA3 Test

By contrast with serum PSA, the PCA3 score did not correlate with prostate volume (Spearman *r* coefficient = −0.0791, *p* = 0.054). The median urinary PCA3 score was significantly higher in the patients with positive biopsies (Table 1). Patients with a PCA3 score ≥35 had a higher risk of positive biopsies: 66% *vs.* 31% (*p* < 0.001); similarly, the risk was significantly higher using a cutoff of 21: 62% *vs.* 22% (*p* < 0.001) (Table 1). The risk of positive biopsies increased with increasing score (Figure S1).

For predicting any PCa, the PCA3 score disclosed an area under the receiver operating curve (AUC) of 0.743 (95%, CI 0.70–0.78) (Figure S2). At the usual cutoff of 35, sensitivity was 63%, with a specificity of 72% and an accuracy of 68% (Table S2). Similar results were obtained for patients in the PSA grey zone (4–10 ng/mL) with an AUC of 0.736 (95%, CI 0.69–0.78). For predicting HGPCa (Gleason score ≥ 7), the PCA3 score AUC was 0.689 (95%, CI 0.64–0.74). Using Epstein criteria to define HGPCa (>T1c, PSA density ≥ 0.15, Gleason score ≥ 7 and/or proportion of invaded cores ≥ 33%) [24], the PCA3 score AUC was 0.728 (95%, CI 0.69–0.77) with a significantly different median (interquartile range, IQR) when compared to non-significant cancers: 51 (26–97) *vs.* 20 (11–48) (*p* < 0.0001).

Median serum PSA was significantly higher in patients with positive biopsies (Table 1). None of the cutoffs, 2.5, 4 and 10 ng/mL, had a significant ability to predict biopsy results (Table 1). The AUC of initial PSA was 0.517 (95%, CI 0.47–0.56), significantly lower than that of the PCA3 score (*p* < 0.0001) (Figure S2). Similarly, at 0.562 (95%, CI 0.50–0.62), it was significantly lower than that of the PCA3 score to predict HGPCa (*p* < 0.001).

Using univariate analysis, age, DRE findings, prostate volume and the PCA3 score, but not serum PSA, were the predictors of any PCa and HGPCa (Table S3). In logistic regression models, all criteria, including serum PSA, achieved independent predictor status and were included in a “base model” (age, DRE findings, prostate volume and serum PSA) and additional models by adding PCA3 as either a continuous or binary variable. We used the widely used cutoff of 35 [9,12] and the recently published cutoff of 21 [14]. We found that the three models, including the PCA3 score, gave significantly higher AUCs (≥0.780) than the base model (0.714) and provided a significantly better accuracy (≥71%) than the base model (66%) in predicting any PCa (Table 2) or HGPCa (Table S4). Decision curve analysis confirmed a higher benefit when adding the PCA3 score (either continuous or binary with a cutoff of 35) to the base model (Figure S3). The nomogram recently published by Hansen *et al.* [14] and specifically proposed for initial prostate biopsy was applied to our whole cohort of 594 patients. Comparison with the actual biopsy results confirmed a strong correlation between prediction and pathological findings (Figure S4): the observed proportion of positive biopsies increased with the calculated PCa risk (*p* < 0.001). The nomogram provided a 70% predicted accuracy and an AUC of 0.764 (95%, CI 0.726–0.802).

### 2.3. Head-to-Head Comparisons of the 3 Available Urinary PCA3-Based Nomograms

**Figure 1:**
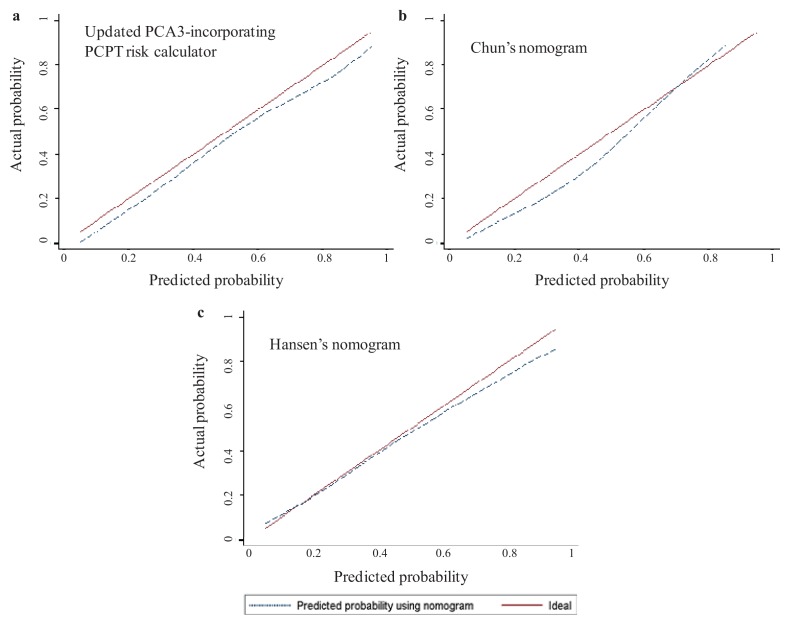
Calibration plots within external validation cohort using the three available urinary PCA3-based nomograms (*n* = 536 patients): (**a**) PCA3, including updated Prostate Cancer Prevention Trial risk calculator [20]; (**b**) Chun’s nomogram [21]; and (**c**) Hansen’s nomogram [14].

**Figure 2:**
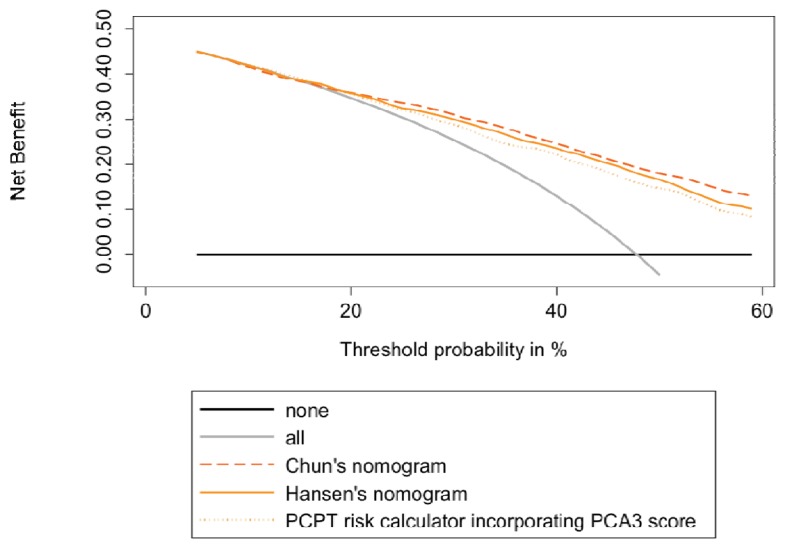
Decision curve analysis of predicting prostate cancer on initial prostate biopsy using the three available urinary PCA3-based nomograms (*n* = 536 patients).

For head-to-head comparison of the three nomograms, we excluded patients <55 (age ≥55 is required for the PCPT risk calculator) and, therefore, evaluated 536 patients. For the three nomograms, AUCs were ≥0.730 and predictive accuracies, ≥67% (Figure S5). No statistically significant difference was observed when comparing these performances. Calibration plot curves are provided in Figure 1, as a representation of the PCa probability predicted by the nomogram and the actual observed proportion of positive initial biopsies. Although it did not attain perfect calibration, Hansen’s nomogram was slightly better calibrated than the two others. To compare the predictive net benefit of the three nomograms, we used decision curve analysis (Figure 2). Chun’s and Hansen’s nomograms disclosed the highest net benefits, while the Hansen one provided the lowest underestimation rate. As expected, a net reduction of unnecessary biopsies was observed using each of the three nomograms, while missing a few HGPCa. When applying nomogram-derived PCa probability thresholds ≤30%, the PCPT risk calculator would have missed only 5% of any PCa and 3% of HGPCa, but only 22% of the biopsies would have been avoided in patients without PCa. Using Chun’s or Hansen’s nomograms, initial biopsy could be avoided up to 48% or 43% of the patients without PCa, respectively, while missing ≤11% of any PCa and only ≤6% of HGPCa (Table 3). Similar results were obtained when defining significant cancers, according to Epstein criteria. Using thresholds ≤ 30% and the PCPT risk calculator, Chun’s and Hansen’s nomograms would have missed three to 7% of significant cancers.

### 2.4. Discussion

Consequent efforts have been made to provide algorithms that could accurately evaluate the actual risk of PCa, while deciding whether prostate biopsies have to be performed. Several studies even disclosed the improvement of predictive accuracy when adding a PCA3 score to a previously published nomogram [25] or incorporating it in a new one [9,12,16–19]. Two urinary PCA3-based nomograms were available for external use. They are not specifically devoted to patients scheduled for initial biopsy, but this medical history has to exist in order to obtain a risk calculation [20,21]. Recently, Hansen *et al.* [14] developed a novel, internally validated, urinary PCA3-based nomogram, specifically for men scheduled for initial prostate biopsies. Similarly to the two others, this nomogram significantly improved the accuracy of biopsy outcome prediction.

To compare these three available nomograms in predicting the outcome of initial prostate biopsies, we took the opportunity to use a large French single-institution patient cohort. We first characterized this cohort and checked that the diagnostic performance of urinary PCA3 test, as assessed using both univariate and multivariate analyses, was quite similar to that observed in previous studies [12–15]. Performance was conserved in the PSA grey zone of 4–10 ng/mL. The PCA3 score proved to be an independent predictor of initial biopsy outcome, as previously observed [6–15]. The addition of the PCA3 score to a base model, including classical prediction factors (age, DRE findings, prostate volume and total PSA), proved, again, a significant increase in predictive accuracy [9,12,16–19]. In our whole cohort of 594 patients, Hansen’s nomogram, which uses the PCA3 score as a binary variable around a cutoff of 21, gave results slightly inferior to that observed in the princeps study. We found an AUC of 0.764 (95%, CI 0.726–0.802) as compared to the reported one: 0.807 (95%, CI 0.768–0.828) [14]. These results, along with those obtained using calibration curves and decision curve analyses (Figures 1 and 2) nevertheless suggest that the present study could be considered as the claimed [26] external validation study of Hansen’s nomogram. Our cohort is, however, monocenter and exclusively composed of French patients. Even if the strong correlation between our results and the published ones underlines the nomogram’s robustness, results have therefore to be generalized with caution until verifications have been performed in other populations.

As a limitation of their study, Hansen *et al.* [26] acknowledged the lack of comparison between their nomogram and other existing predictive tools. Direct comparison between Chun’s nomogram and the PCA3-updated PCPT risk calculator has previously been performed in a cohort of 218 patients scheduled for either initial or repeat biopsies [27]. The two nomograms were found to be equivalent in terms of predictive accuracy, proportion of saved biopsies and proportion of missed cancers, but Chun’s nomogram provided better overall calibration and a higher net benefit on decision curve analyses. In the present study, although we found no statistical differences between the three nomograms when comparing AUCs and predictive accuracies, we observed a trend towards the lessened diagnostic performance of the PCPT risk calculator. This was also underlined using both calibration plot curves and decision curve analyses. The reasons why PCPT seems less accurate remain to be determined, but at least two intrinsic particularities can be noticed: the PCA3 score is used as a continuous variable, and prostate volume is not included as a prediction factor. Using the PCA3 score as a binary variable around a cutoff seems indeed to display a better predictive accuracy [12,14,21]. Moreover, Hansen *et al.* [14] considered as crucial the inclusion of prostate volume within their nomogram, and this criteria was also part of Chun’s one [21]. When checking the application of the urinary PCA3 test in a multivariate regression model, we also found prostate volume to be an independent predictor of biopsy outcome. It is worthy to note that the PCA3 thresholds used in our study and the previously published ones are not always the same: 17 for Chun *et al.* [21], 21 for Hansen *et al.* [14] and 35 for de la Taille *et al.* [12] and our study. The FDA retained the threshold of 25 for men subjected to repeat biopsy. No consensual threshold is currently available for men subjected to initial biopsy, but it could be assumed that the lower one is the chosen threshold; the lowest being the risk of missing (significant) PCas.

One could question the head-to-head analyses between Hansen’s nomogram and nomograms not specifically devoted to the initial biopsy decision. However, the fact that Chun’s nomogram provided the best AUC and predictive accuracy suggests that they can reliably be compared. In this regard, calibration plot curves and decision curve analyses, of strong interest when comparing diagnostic markers and nomograms, also showed that comparison is not as biased as expected, since the higher net benefit was observed with Chun’s nomogram (Figure 2), while Hansen’s one provided the calibration curve nearest to the perfect prediction diagonal (Figure 1). These analyses showed that application of either nomogram to our cohort would have induced a reduction in the number of prostate biopsies performed, although lesser than expected, as confirmed by Table 3. Very similarly to the reported one, the increase in predictive accuracy is about 6% when adding PCA3 and can be considered, together with the intrinsic value of predictive accuracy (about 70%), as poorly significant in clinical practice. However, we found that at a probability threshold of 30%, Chun’s and Hansen’s nomogram would have saved 30% and 27% of biopsies, respectively, while missing only 6% of HGPCa, as defined by a biopsy Gleason sum ≥7. These results are quite similar to that previously observed in Hansen’s princeps paper (36% of saved biopsies and 2% of missed HGPCa) and in the head-to-head comparison between the two other available PCA3-based nomograms [27]. While Chun’s nomogram [21] would have saved 28% of biopsies, while missing 3.7% of HGPCa, PCA3 including the PCPT risk calculator [20] would have saved 18% of biopsies, while missing no HGPCa [27]. We mainly used in the present paper the definition of HGPCa used by the authors of the nomograms [14,21], that is, the one only based on a Gleason sum ≥7. In fact, up to 50% of the patients with a biopsy Gleason sum at six eventually disclosed upgrading to a Gleason of seven or more at prostatectomy [28]. Of interest, when applying Epstein criteria to identifying significant cancers [24], the diagnostic performance of the PCA3 score (AUC = 0.728) was at least as high as that observed when using a Gleason sum as the sole criterion (AUC = 0.689). It is likely that the good performance of the PCA3 test using the Epstein criteria is related to the fact that this classification takes into account tumor volume estimation, a variable known to be predicted by the PCA3 score [29]. In our experience, PCA3-incorporating nomograms also reduce the number of useless biopsies without missing more than 7% of Epstein-defined significant PCas, a result underlying PCA3 test robustness.

## 3. Experimental Section

### 3.1. Patients and Study Design

Between January, 2008, and January, 2013, consecutive patients scheduled in the Department of Urology of our secondary institution were included to have an initial prostate biopsy, because of elevated serum PSA (≥4 ng/mL) and/or suspicious digital rectal examination (DRE), whatever the PSA levels. Patients with serum PSA ≥20 ng/mL were eventually excluded. The institutional review board approved this study, and all patients provided informed written consent to participate. Excluded were patients with previous prostate biopsy (biopsies) (whatever the results), any medical therapy affecting serum PSA levels and/or previous prostate surgery for benign hypertrophy (BPH).

### 3.2. Biochemical Assays and Nomograms

After standardized DRE and before initial prostate biopsy, the first voided urine was collected as previously described, and PCA3 and PSA RNA quantification was performed as recommended using the Progensa™ PCA3 Assay (Hologic^®^ Gen-Probe) [29,30]. The three nomograms used are summarized in Table 4. The updated PCPT calculator risk, including the PCA3 score [20], is available online (http://deb.uthscsa.edu/URORiskCalc/Pages/calcs.jsp), allowing PCa risk calculation. The probability of PCa was graphically calculated from Chun’s [21] and Hansen’s nomograms [14].

### 3.3. Prostate Biopsies

Prostate volume was measured by TRUS (*trans*-rectal ultrasonography) using the elliptical formula. A TRUS-guided prostate biopsy was performed with at least 12 cores (there are possible additional cores from suspicious areas according to DRE and/or TRUS findings). Histological examinations were performed according to international standards by experienced pathologists unaware of the PCA3 test results.

### 3.4. Statistics

Continuous variables were expressed as medians and interquartile ranges (IQR). They proved to be normally distributed using the Skewness and Kurtosis test, except for patient age. The parametric Student’s *t*-test and non-parametric Wilcoxon *U* test were performed to determine differences for continuous variables. The chi-square test was used to compare proportions. Univariate and multivariate logistic regression models addressed the presence of any PCa at the initial prostate biopsy. Areas under receiver operating curves (AUC) were used to address discriminative properties of the tested variables and to identify the best cutoff, compared using Hanley’s test. Predictive accuracies were defined as the proportion of patients correctly classified using an individual marker or the regression models. The extent of over- and under-estimation of the observed *vs.* predicted PCa probability was explored graphically using regression smoothing plot curves. The relationship between the threshold probability of biopsy outcome and the relative value of false-positive and false negative results was examined using decision curve analyses to determine the net benefit of the predictive models [31]. Data were analyzed using software package STATA^®^v11.0 (College Station, TX, USA), with *p* <0.05 considered to be statistically significant.

## 4. Conclusions

In conclusion, we provided valuable data to consider the urinary PCA3 score as a useful adjunct to predict the results of initial prostate biopsies. Either Chun’s (dedicated to either initial or repeat biopsies) or Hansen’s nomograms can be considered of added value when considering the issue of initial prostate biopsy. Conversion of these nomograms into an online available calculator is likely to be beneficial in clinical practice.

## Supplementary Information



## Figures and Tables

**Table 1:** Patient characteristics and initial biopsy results (*n* = 594).

	Entire initial biopsy cohort	No cancer at initial biopsy	LGPCa at initial biopsy	HGPCa at initial biopsy	*p*-Value *
No. of patients (%)	594 (100)	318 (54)	148 (25)	128 (22)	-
Age, year					<0.0001
Median	63	62	64	65	
IQR	58–67	58–66	59–68	61–70	
DRE					<0.001
Unsuspicious, no. (%)	519 (87)	293 (92)	136 (92)	90 (70)	
Suspicious, no. (%)	75 (13)	25 (8)	12 (8)	38 (30)	
Familial history of PCa					0.428
No (%)	505 (85)	273 (86)	121 (82)	111 (87)	
Yes (%)	89 (15)	45 (14)	27 (18)	17 (13)	
Prostate volume *, mL					<0.0001
Median	39.5	42	37	30	
IQR	28–50	32–55	29–48	24–47	
Serum PSA, ng/mL					0.012
Median	5.9	6	5.7	6.1	
IQR	4.7–7.9	4.6–7.6	4.6–7.4	5.1–8.9	
≥2.5 ng/mL	579 (97)	308 (97)	143 (97)	128 (100)	0.119
≥4 ng/mL	525 (88)	279 (88)	133 (90)	113 (88)	0.799
≥10 ng/mL	70 (12)	32 (10)	17 (11)	21 (16)	0.170
Urinary PCA3 score					<0.0001
Median	30	18	48	52	
IQR	15–65	10–42	25–94	26–100	
≥35	265 (45)	90 (28)	93 (63)	82 (64)	<0.001
<35	329 (55)	228 (72)	55 (37)	46 (36)	
>21	364 (61)	138 (43)	117 (79)	109 (85)	<0.001
≤21	230 (39)	180 (57)	31 (21)	19 (15)	

PCa = prostate cancer; DRE = digital rectal examination; LGPCa = low-grade PCa; HGPCa = high-grade PCa (Gleason score ≥ 7); PCA3 = prostate cancer antigen 3; PSA = prostate-specific antigen; IQR = interquartile range;

* comparison of the three groups: no cancer, LGPCa and HGPCa.

**Table 2:** Multivariate analysis evaluating performance of logistic regression models to predict initial prostate biopsies.

	Multivariate analysis							
	
	Base model		Base model + continuous PCA3 score		Base model + PCA3 cutoff of 21		Base model + PCA3 cutoff of 35	
	
	OR (95% CI)	*p*-Value	OR (95% CI)	*p*-Value	OR (95% CI)	*p*-Value	OR (95% CI)	*p*-Value
Age, year	1.08 (1.05–1.11)	<0.001	1.05 (1.02–1.09)	<0.001	1.06 (1.02–1.09)	<0.001	1.05 (1.02–1.08)	0.001
DRE	1.09 (1.03–1.15)	0.004	1.08 (1.02–1.15)	0.006	1.08 (1.02–1.15)	0.008	1.09 (1.03–1.16)	0.004
Prostate volume, cm^3^	0.96 (0.95–0.97)	<0.001	0.96 (0.95–0.97)	<0.001	0.96 (0.95–0.98)	<0.001	0.96 (0.95–0.97)	<0.001
Serum PSA, ng/mL	1.10 (1.03–1.17)	0.004	1.10 (1.02–1.18)	0.008	1.08 (1.01–1.16)	0.027	1.09 (1.02–1.17)	0.015
Urinary PCA3 score	-	-	1.01 (1.01–1.01)	<0.001	5.00 (3.36–7.45)	<0.001	4.21 (2.88–6.15)	<0.001
AUC	0.714		0.780		0.781		0.780	
IC 95%	(0.672–0.755)		(0.743–0.818)		(0.744–0.818)		(0.742–0.817)	
*p-*Value *	-		*p* < 0.0001		*p* < 0.0001		*p* < 0.0001	
PA	66%		72%		71%		73%	
IC 95%	(62%–70%)		(68%–76%)		(68%–75%)		(69%–76%)	
Increment in PA *	-		+6%		+5%		+7%	
*p*-Value *	-		*p* = 0.033		*p* = 0.060		*p* = 0.017	

AUC = area under the receiver operating curve; CI = confidence interval; DRE = digital rectal examination (suspicious *vs.* unsuspicious); OR = odds ratio; PA = predictive accuracy (proportion of well-classified patients according to the best automatically calculated cutoff); PSA = prostate-specific antigen; PCA3 = prostate cancer antigen 3;

* when comparing to the base model.

**Table 3:** Numbers of biopsies performed and detection rates of any prostate cancer and high-grade prostate cancer (Gleason score ≥ 7), according to the three urinary PCA3-based nomograms-derived probability cut-offs.

Nomogram	Probability cutoff (%)	Biopsies performed	Biopsies not performed a	Biopsies not performed in men without PCa b	Any PCa detected c	Any PCa missed	NPV for PCa prediction	HGPCa detected	HGPCa missed	NPV for HGPCa prediction
		*n* (%)	*n* (%)	*n* (%)	*n* (%)	*n* (%)	%	*n* (%)	*n* (%)	%
	None	536 (100)	0 (0)	0 (0)	256 (100)	0 (0)	100	122 (100)	0 (0)	100

PCPT [20]	10	531 (99)	5 (1)	5 (2)	256 (100)	0 (0)	100	122 (100)	0 (0)	100
	20	512 (96)	24 (4)	22 (8)	254 (99)	2 (1)	92	121 (99)	1 (1) *	98
	30	462 (86)	74 (14)	66 (22)	244 (95)	12 (5)	84	118 (97)	4 (3) *	95
	40	376 (70)	160 (30)	123 (44)	219 (86)	37 (14)	77	108 (89)	14 (11) **	93
	50	275 (51)	261 (49)	184 (66)	179 (70)	77 (30)	71	90 (74)	32 (26) ***	88

Chun [21]	10	516 (96)	20 (4)	17 (6)	253 (99)	3 (1)	85	122 (100)	0 (0)	100
	20	462 (86)	74 (14)	63 (23)	245 (96)	11 (4)	85	121 (99)	1 (1) *	98
	30	375 (70)	161 (30)	134 (48)	229 (89)	27 (11)	83	115 (94)	7 (6) *	96
	40	342 (64)	194 (36)	154 (55)	216 (84)	40 (16)	79	108 (89)	14 (11) *	93
	50	249 (46)	287 (54)	204 (73)	173 (68)	83 (32)	71	93 (76)	29 (24) ****	90

Hansen [14]	10	524 (98)	12 (2)	11 (4)	255 (99.6)	1 (0.4)	92	122 (100)	0 (0)	100
	20	466 (87)	70 (13)	66 (22)	248 (97)	8 (3)	89	121 (99)	1 (1) *	98
	30	390 (73)	146 (27)	119 (43)	229 (89)	27 (11)	82	115 (94)	7 (6) *	96
	40	345 (64)	191 (36)	149 (53)	214 (84)	42 (16)	78	108 (89)	14 (11) *	93
	50	292 (54)	244 (46)	179 (64)	191 (75)	65 (25)	73	97 (80)	25 (20) **	90

PCa = prostate cancer; NPV = negative predictive value; HGPCa = high-grade prostate cancer (Gleason score ≥ 7);

a biopsies that would have not been performed if the test, considered negative under the corresponding probability cutoff, has been used to decide biopsy or not;

b part of the number of biopsies not performed (see ^a^) in the subgroup of patients in whom biopsies were eventually revealed to be negative; percentage is indicative of specificity;

c percentage is indicative of sensitivity;

* all men had a Gleason score = 7;

** two men had a Gleason score of 4 + 4; the other men had a Gleason score = 7;

*** one man had a Gleason score of 5 + 4; three men had a Gleason score of 4 + 4; the other men had a Gleason score = 7;

**** three men had a Gleason score of 4 + 4; the other men had a Gleason score = 7.

**Table 4:** The three compared urinary PCA3-based nomograms used in the present study.

	Race a	Age (year)	Serum PSA (ng/mL)	Family history (yes/no)	DRE e	Negative previous biopsy(ies) (yes/no)	Prostate volume (cm^3^)	Urinary PCA3 score f
PCPT [20]	+	+ b	+	+	+	+		+
Chun [21]		+	+ c		+	+	+	+
Hansen [14]		+	+ d		+		+	+

a African American, Caucasian, Hispanic or other (accounting only for HGPCa risk calculation);

b ≥55 years (younger patients are excluded);

c ≤50 ng/mL;

d ≤20 ng/mL;

e suspicious *vs.* unsuspicious;

f Progensa^®^ PCA3 assay. A “+” is indicated when the corresponding criterion (race, age, PSA…) is used to calculate the prostate cancer risk in the nomogram.
