# Supplementary material for: PCA3 and PCA3-Based Nomograms Improve Diagnostic Accuracy in Patients Undergoing First Prostate Biopsy
Source: Int J Mol Sci. 2013 Aug 29;14(9):17767–80. doi: 10.3390/ijms140917767 (PMC3794752; doi:10.3390/ijms140917767)
Supplement: Supplementary file 1 [file ijms-14-17767-s001.pdf]

Supplementary Information

Figure S1. Risk of positive initial biopsies depending on urinary PCA3 score (*n* = 594 patients) Numbers of patients are indicated below the histograms for each category of PCA3 score. PCA3 = prostate cancer gene 3; PCa = prostate cancer.

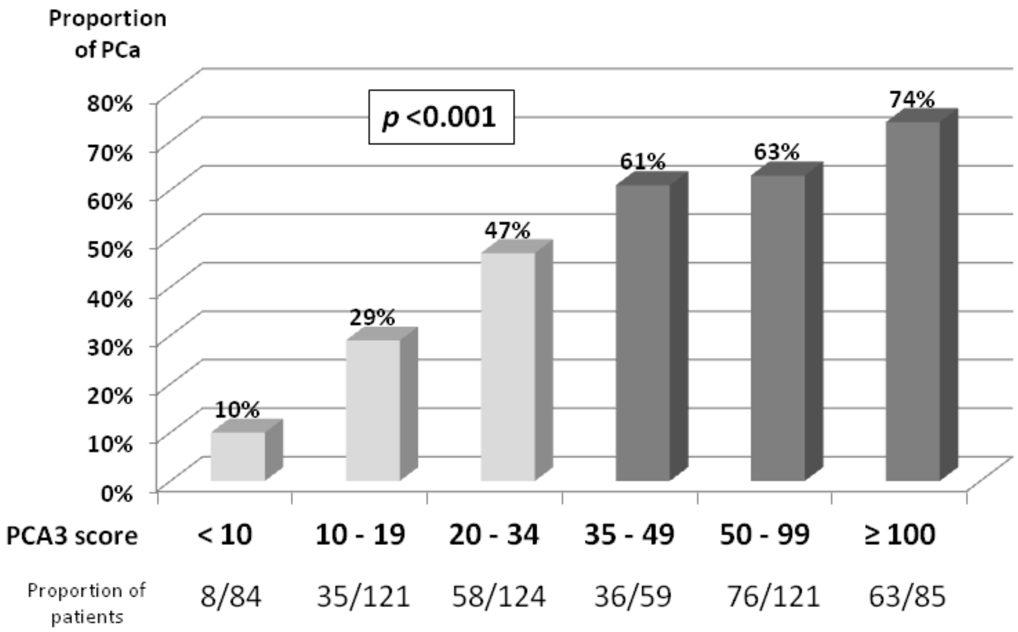

Figure S2. Diagnostic performances of serum PSA and urinary PCA3 score in predicting initial biopsy outcome (*n* = 594 patients). PSA = prostate-specific antigen; PCA3 = prostate cancer gene 3.

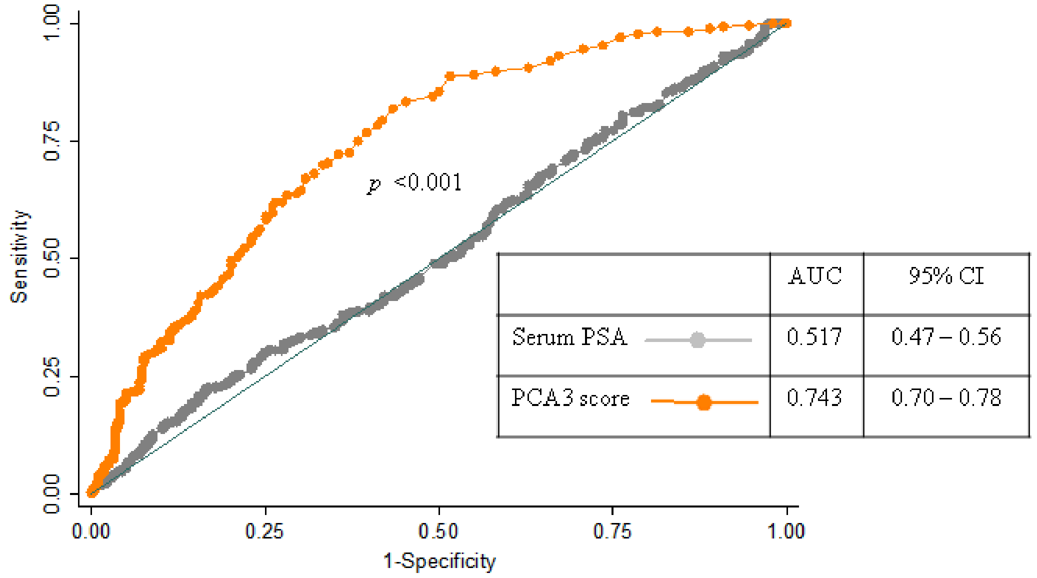

Figure S3. Decision curve analysis of predicting prostate cancer on initial prostate biopsy using regression models (*n* = 594 patients). Base model included age, DRE findings (suspicious *vs*. non-suspicious), prostate volume and serum total PSA. Urinary PCA3 score was added to the base model as either a continuous or a binary variable (around a cutoff 35) variable.

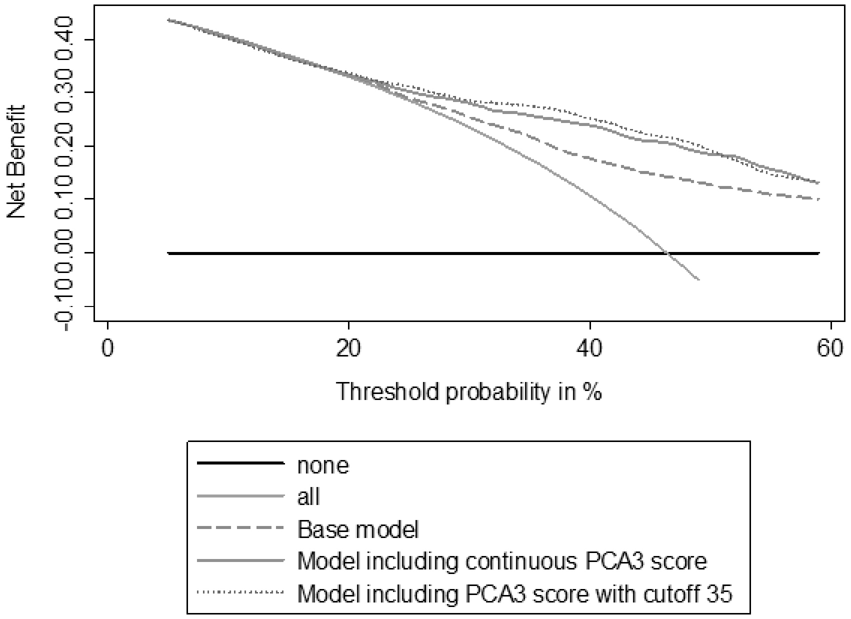

Figure S4. Comparison between the risk of prostate cancer as predicted by the Hansen's nomogram and the actual proportion of positive initial biopsies in the entire population (*n* = 594 patients). PCa = prostate cancer.

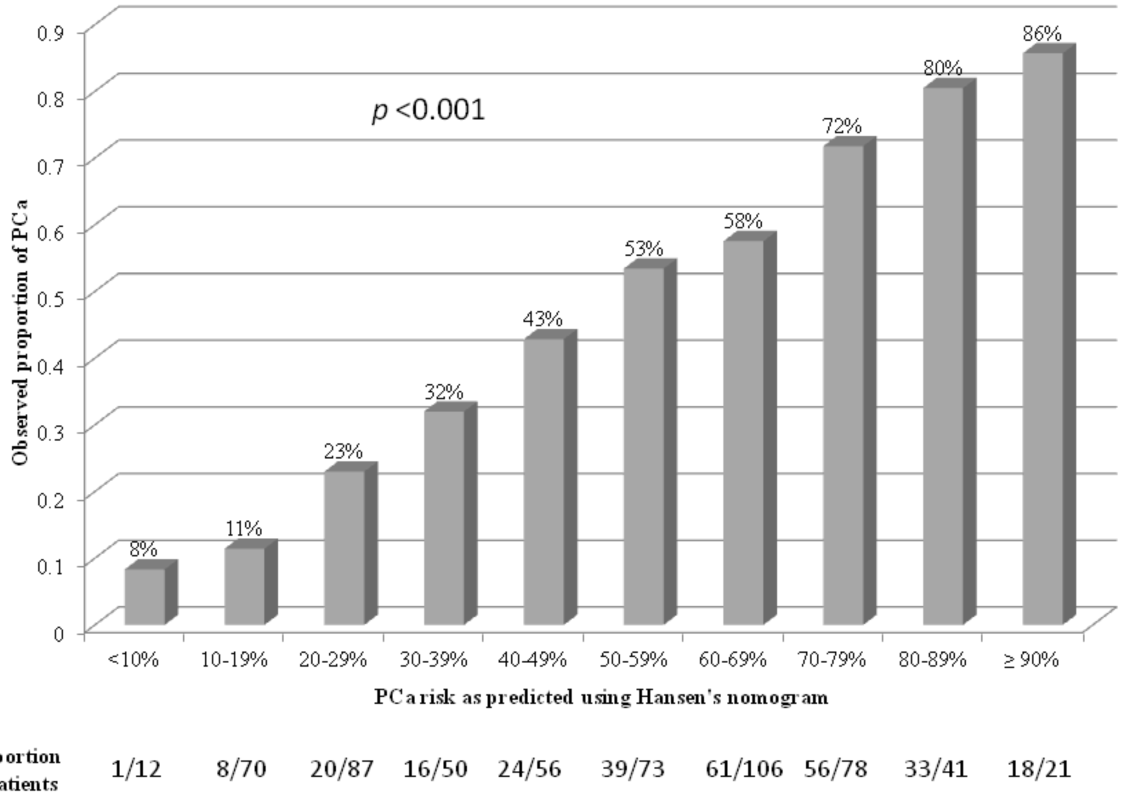

Figure S5. Comparison of performances of the three published urinary PCA3-incorporating nomograms in predicting results of initial prostate biopsies (*n* = 536 patients) AUC = area under the curve; CI = confidence interval; PCPT = PCA3-incorporating prostate cancer prevention trial risk calculator. * Proportion of well-classified patients according to the best automatically calculated cutoff.

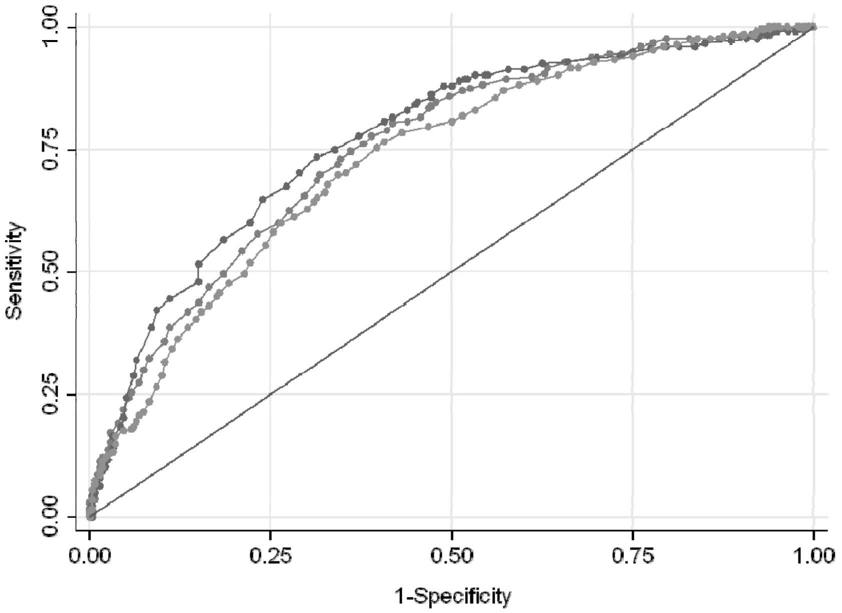

	AUC (95% CI)	Predictive accuracy* (95% CI)
Updated PCA3-incorporating PCPT Risk calculator [20]	0.730 (0.688 – 0.772)	66.6% (62.6 – 70.6)
Hansen's nomogram [14]	0.753 (0.713 – 0.794)	69.0% (65.1 – 73.0)
Chun's nomogram [21]	0.773 (0.733 – 0.812)	70.3% (66.4 – 74.2)

$p = 0.053$ (Updated PCA3-incorporating PCPT Risk calculator vs. Chun's nomogram); $p = 0.201$ (Updated PCA3-incorporating PCPT Risk calculator vs. Hansen's nomogram); $p = 0.115$ (Hansen's nomogram vs. Chun's nomogram)

Table S1. Pathological findings.

**Pathological findings**			
Number of sampled cores [a]			
	12 cores	*n* = 520	88%
	13–14 cores	*n* = 64	11%
	15–18 cores	*n* = 10	2%
Gleason score [b]			
	3 + 3 = 6	*n* = 148	54%
	3 + 4 = 7	*n* = 81	29%
	4 + 3 = 7	*n* = 30	11%
	4 + 4 = 8	*n* = 15	5%
	4 + 5 = 9	*n* = 1	0.5%
	5 + 4 = 9	*n* = 1	0.5%
Proportion of invaded cores [b]			
	≤33%	*n* = 191	69%
	>33%	*n* = 85	31%
Proportion of invaded tissue [b,c]			
	Median (IQR)	5%	(2%–12%)

[a] Assessed in the 594 patients; [b] Assessed in the 276 patients with positive biopsies; [c] Ratio length of invaded prostatic tissue/total length of biopsied prostatic tissue; IQR: interquartile range.

Table S2. Variation of diagnostic performances of urinary PCA3 test depending on various cutoffs.

**Cutoff**	**Sensitivity**	**Specificity**	**Positive predictive value**	**Negative predictive value**	**Accuracy**
17	89%	45%	58%	83%	65%
21	82%	57%	62%	78%	68%
24	78%	59%	62%	76%	68%
30	70%	67%	65%	72%	68%
35	63%	72%	66%	69%	68%
40	60%	74%	67%	68%	67%
45	55%	77%	67%	66%	67%
50	50%	79%	67%	65%	66%

PCA3 = prostate cancer gene 3; accuracy = proportion of correctly classified patients.

Table S3. Univariable logistic regression models predicting any prostate cancer and high-grade prostate cancer at initial biopsy.

	**Any PCa**				**HGPCa***			
	**OR (95% CI)**	**p-Value**	**AUC (95% CI)**	**PA**	**OR (95% CI)**	**p-Value**	**AUC (95% CI)**	**PA**
Age, year	1.05 (1.03–1.08)	<0.001	0.602 (0.556–0.647)	58.1%	1.07 (1.04–1.11)	<0.001	0.624 (0.570–0.679)	78.5%
DRE (suspicious *vs.* unsuspicious)	1.10 (1.05–1.16)	<0.001	0.690 (0.649–0.705)	57.7%	1.18 (1.12–1.24)	<0.001	0.742 (0.736–0.748)	78.6%
Prostate volume, cm^3	0.97 (0.96–0.98)	<0.001	0.641 (0.597–0.685)	60.8%	0.97 (0.96–0.98)	<0.001	0.651 (0.595–0.708)	78.5%
Serum PSA, ng/mL	1.03 (0.98–1.09)	0.257	0.517 (0.470–0.563)	54.0%	1.09 (1.03–1.16)	0.004	0.562 (0.504–0.620)	78.5%
PCA3 score, continuously coded	1.01 (1.01–1.02)	<0.001	0.743 (0.704–0.782)	63.6%	1.01 (1.00–1.01)	<0.001	0.689 (0.641–0.736)	77.4%
PCA3 score >21 *vs.* ≤21	5.90 (4.04–8.61)	<0.001	0.794 (0.793–0.795)	68.4%	4.75 (2.82–7.99)	<0.001	0.785 (0.781–0.788)	78.5%
PCA3 score ≥35 *vs.* <35	4.39 (3.11–6.20)	<0.001	0.743 (0.741–0.745)	67.9%	2.76 (1.84–4.14)	<0.001	0.661 (0.613–0.701)	78.5%

Analyses were performed in the 594 patients. PCa = prostate cancer; HGPCa = high-grade prostate cancer (Gleason score ≥7); OR = odds ratio; CI = confidence interval; AUC = area under the curve; PA = predictive accuracy using the best calculated cutoff; PSA = prostate-specific antigen; DRE = digital rectal examination; PCA3 = prostate cancer gene 3; * For this analysis, men with low-grade prostate cancer (Gleason score <7) were classified the same as men with negative biopsies.

Table S4. Multivariate analysis evaluating performances of logistic regression models to predict high-grade prostate cancer.

	**Multivariate analysis**							
	**Base model**		**Base model+ continuous PCA3 score**		**Base model+ PCA3 cutoff 21**		**Base model+ PCA3 cutoff 35**	
	OR (95% CI)	*p*-Value	OR (95% CI)	*p*-Value	OR (95% CI)	*p*-Value	OR (95% CI)	*p*-Value
Age, year	1.09 (1.05–1.12)	<0.001	1.08 (1.04–1.11)	<0.001	1.07 (1.03–1.11)	<0.001	1.07 (1.03–1.11)	<0.001
DRE	1.17 (1.10–1.24)	<0.001	1.17 (1.10–1.24)	<0.001	1.17 (1.10–1.24)	<0.001	1.17 (1.11–1.24)	<0.001
Prostate volume, cm^3	0.95 (0.94–0.97)	<0.001	0.96 (0.94–0.97)	<0.001	0.96 (0.94–0.97)	<0.001	0.96 (0.94–0.97)	<0.001
Serum PSA, ng/mL	1.16 (1.08–1.25)	<0.001	1.16 (1.07–1.25)	<0.001	1.15 (1.06–1.24)	<0.001	1.15 (1.07–1.24)	<0.001
Urinary PCA3 score	-	-	1.00 (1.00–1.01)	0.003	3.62 (2.07–6.36)	<0.001	2.3 (1.46–3.64)	<0.001
AUC	0.770		0.788		0.797		0.791	
IC95%	(0.723–0.817)		(0.744–0.833)		(0.754–0.839)		(0.747–0.834)	
p-Value*	-		*p* = 0.036		*p* = 0.037		*p* = 0.049	
PA	81.3%		81.1%		81.5%		81.8%	
IC 95%	(78.2%–84.4%)		(78.0%–84.3%)		(78.3%–84.6%)		(78.7%–84.9%)	
Increment in PA*	-		−0.2%		+0.2%		+0.5%	
p-Value *	-		*p* = 0.941		*p* = 0.941		*p* = 0.822	

Analyses were performed in the 594 patients. AUC = area under the receiver operating curve; CI = confidence interval; DRE = digital rectal examination (suspicious vs. unsuspicious); OR = odds ratio; PA = predictive accuracy (proportion of well-classified patients according to the best automatically calculated cutoff); PSA = prostate-specific antigen; PCA3 = prostate cancer gene 3. * when comparing to the base model.

© 2013 by the authors; licensee MDPI, Basel, Switzerland. This article is an open access article distributed under the terms and conditions of the Creative Commons Attribution license (http://creativecommons.org/licenses/by/3.0/).
